# Experimental and coupling analysis of municipal solid waste (MSW) shear strength under multiple influencing parameters

**DOI:** 10.1371/journal.pone.0344191

**Published:** 2026-03-05

**Authors:** Muhammad Muneer, Dequan Kong, Rong Wan, Xinyang Liu, Qingxiang Song, Masood ur Rahman

**Affiliations:** School of Civil Engineering, Chang’an University, Xi’an, China; University of the West of England, UNITED KINGDOM OF GREAT BRITAIN AND NORTHERN IRELAND

## Abstract

The production of municipal solid waste (MSW) in China is growing rapidly, and landfill is the primary treatment method. The shear strength of MSW is crucial to the stability and safety of landfills. However, there is limiting research available on how different environmental and compositional factors interactively influence MSW shear strength since some existing studies typically examine these variables in isolation. Through integrated testing and statistical modeling this looks into how environmental factors (moisture content 30–230%, organic matter content 15–75%, temperature 10–65°C and degradation time 15–190days) work together to change the shear strength of MSW. One hundred MSW samples were prepared according to field waste composition from Xi’an and were subjected to direct shear tests over a 190-day degradation period. The test results show that cohesion (*c*) decreased over time, to a minimum value of 0.17 kPa at 190 days, while angle of internal friction (*φ*) increased to 42.39° at 190 days. Both *c* and *φ* showed an initial increase followed by a decrease with temperature changes. The effect of organic matter on *c* decline was negligible, though *φ* increased during the early stage of degradation. Higher moisture content (80–130%) and organic matter (45–60%) along with temperatures between 10–35°C resulted in high *φ* values. High *c* values were achieved with 130% moisture content 15–30% organic matter and temperatures between 10 and 35 °C. Under the controlled conditions of the study the Mohr–Coulomb equation was modified to incorporate degradation effects, and multiple linear regression together with automatic linear modeling (ALM) were used to predict *c* and *φ*, yielding R^2^ values of 0.622 and 0.468 respectively, demonstrating the models’ predictive accuracy. In practical engineering, the study’s outcomes will help to understand the effect of different parameters on the shear strength of MSW which can lead to achieve the landfill slope stability and the use of MSW as backfill material in building foundations under the controlled conditions of the study.

## 1. Introduction

Over the past few decades, municipal solid waste (MSW) generation has become a critical global environmental issue due to rapid urbanization and industrial growth. The improper management of MSW can lead to problems such as land degradation, greenhouse gas emissions, and public health risks. Due to insufficient MSW recycling facilities developing regions like China and Middle East are facing growing challenges of waste management [1,2]. According to the recent estimates the global daily production of MSW is about to 3.3 million tons and is expected to increase to 11 million tons per day by the end of the twentieth century [3]. China generated more than 235 million tons of municipal solid waste in 2020 and was expected to reach the annual output will reach 280 million tons by 2025 [4]. The global MSW output is projected to increase to 4 billion tons by 2050 [5]. This robust increase in urban solid waste generation has brought serious environmental and climate change challenges especially greenhouse gas emissions from landfills [6,7]. Some researchers have also studied the scope of using of waste materials in concrete and construction materials to find sustainable solutions and to reduce the overall volume of waste materials [8]. However, sanitary landfilling remains the most common method of MSW disposal in many regions, including China [9]. There is a risk of landfill collapse as the slope steepens and the pile height increases with the amount of waste deposited [10]. Under conditions of increased waste height and high moisture content the little shear strength in MSW can cause the slope failures, uneven settlement and landfill instability. In geotechnical engineering the shear strength parameters *c* and *φ* directly govern the factor of safety of landfills, making them key to both effective MSW management and landfill stability. Considering that MSW is not only the treatment target of landfills but also the construction and composition material of landfills, the shear strength of MSW is decisive for the stability and safety of landfills [11,12]. Nowadays, urban domestic waste is increasingly used as backfill material for building foundations, making it particularly important to conduct a more scientific evaluation of its shear strength characteristics [13].

Several researchers conducted shear strength experiments on municipal solid waste and found that the shear strength of municipal solid waste increases with the age of landfills [14,15]. However, other experts believe that the shear strength of urban solid waste decreases as landfills age [16]. According to specific experts, the *c* rises while the *φ* falls with landfill age [17], while Others argue that as landfills age, the *c* value decreases while the *φ* value increases. [18,19]. Mohammad et al., [20], claimed that the chemical properties of municipal solid waste degrade rapidly over time, thus stabilizing landfills. Previous studies have shown that the shear strength of municipal solid waste is affected by a variety of factors, including waste type, composition, degree of decomposition, and moisture content [21]. He et al., [22] found that the c-value of municipal solid waste (MSW) increased while the *φ*-value decreased with increasing moisture content. In contrast, Zhao et al., [23] reported that the *φ*-value increased while the *c*-value first increased and then decreased with increasing moisture content. Pulat [24] noted that the waste composition and operating procedures at a particular site are closely related to the shear strength and density of the waste. Reddy et al., [17] studied how moisture content affects the biodegradation of municipal solid waste and found that the cohesive force of fresh municipal solid waste ranged from 31 to 64 kPa, while the drainage friction angle ranged from 26 to 30°. Vilar et al., [25] found that saturation did not affect the compression of municipal solid waste.

Furthermore, landfill temperature affects the stability of municipal solid waste landfills [26]. Weather, water level, waste type, and degree of biodegradation all significantly increase landfill temperature [27]. According to Falamaki et al., [28], increases in temperature can harm landfill stability by decreasing its shear strength. But the impact of temperature on MSW landfill stability under static settings has not been extensively studied [29]. Shi et al., [30] found that cohesiveness decreased linearly with temperature, even as the *φ* remained relatively constant and the maximum temperature increased from 20°C to 50°C. The mechanical characteristics of non-degradable waste components are more significantly impacted by temperature than organic decomposition [31]. A temperature range of 22°C to 45°C is considered for promoting the biodegradation of waste [23]. Singh, V., & Uchimura, T. [32] have discussed how the MSW’s compressibility drops as the proportion of organic materials rises. Compared to the mobilized cohesion, the mobilized angle of internal friction appears to be more strongly affected by degradation. Gradual attenuation of reinforcing materials (such as leather, wood, fabric, and plastics) can lead to slight changes in bond strength [33]. Moisture and organic matter content directly affect the long-term mechanical properties of MSW [34] because they influence the biodegradation process.

In recent years, new experimental setups and modelling methodologies have increased interest in municipal solid waste (MSW) thermo-hydraulic-mechanical coupling (THM) behavior. Using modified large oedometer at temperatures of 25, 45, and 65 °C, Khaleghi et el., [35] have found that the MSW compressibility has increased with rising temperature and the permeability of samples declined with increasing temperature and plastic content. Yazdanpour et al. [36] investigated that in temperature-controlled isotropic compression tests, temperature has enhanced the compression and creep in MSW while decreases elastic response and lowers permeability. Khalghi et al. [37] used an upgraded large-scale consolidation device with temperature control to test MSW compressibility at high temperatures. The waste thermally softened between 25 and 65°C with a higher compressibility index and strain and a decrease in permeability. Machado et el., [38] have modeled thermo-mechanical behavior of waste to predict the mechanical behavior of waste in terms of deviatoric stress, pore water pressure, and volumetric strains in drained and undrained triaxial tests. Yazdanpour et al. [39] performing triaxial shear tests found that the shear strength of MSW is reduced significantly with increasing temperature, with a strength loss of up to approximately 55% at 95°C.

The current study is based on a comprehensive understanding of the field by performing a comprehensive review by following the guidelines of systematic reviews [40,41]. To comply with the PRISMA 2020 guidelines, the study conducted targeted literature searches using major scientific databases such as Scopus, Web of Science, and Google Scholar. To find research on the geotechnical engineering properties of MSW, search keywords included “municipal solid waste,” “shear strength,” “landfill stability,” “moisture content,” “organic matter,” and “temperature.” The initial search yielded approximately 230 articles published between 2000 and 2025. After removing duplicates and articles irrelevant to the geotechnical engineering properties or experimental research of MSW, 42 relevant papers were ultimately included for in-depth analysis. This systematic literature review constructed a fundamental information database and provided guidance for the experimental design and parameter selection in this study.

Several authors have examined the landfill’s shear strength and achieved rich and meaningful results. Still, less research has been conducted on how various dynamic conditions affect the MSW landfill’s shear strength and how they interact with the engineering characteristics of the MSW [42,43]. The planning, construction, and maintenance of landfills, and the use of MSW as backfill material in building foundations, need to take into account the engineering characteristics of MSW [44,45]. However, the shear strength of MSW is a complex issue that is dynamically influenced by multiple parameters, and selecting appropriate shear strength parameters for a site-specific situation remains a challenging engineering design problem [46,47]. The temperature, time (MSW age), moisture content, and organic matter content parameters not only affect MSW strength but also interact with one another, thereby coupling to influence MSW strength [48,49]. The dynamic coupling of MSW strength has received relatively little attention in previous studies, especially in the northwest region of China. Economic development in such areas is rapid, with significant increases in MSW production and complex, diverse composition changes; the scale and number of landfills are constantly increasing, and the engineering problems they face are becoming increasingly urgent. Research on the MSW strength variation law under the coupling impact of several factors is therefore both required and crucial. The study’s primary goals are to understand how dynamic parameters affect MSW’s shear strength behavior, forecast the ideal conditions for high shear strength of MSW under the controlled conditions of the study. Using the statistical program SPSS-2022, the study has developed automatic linear and regression models to forecast the shear strength of MSW under coupled dynamic conditions. The study’s findings on the strength concerns pertaining to the establishment and operation of MSW dumps in adjacent or comparable locations may be referred to and used to guide the future studies.

## 2. Materials and methods

The samples were manually prepared based on the compositional results of MSW research in Xi’an City [50,51]. Before conducting the laboratory experiments, MSW samples were collected from seven representative sites across Xi’an City to reflect real waste conditions and facilitate indoor testing. Table 1 shows the mass percentages of the various MSW compositions in the field-collected samples. The moisture content and density characteristics of MSW in Xi’an are shown in Table 2.

**Table 1:** Mass percentage (%) of MSW components in Xi’an city.

Refuse collection point	Sludge	Food waste	Paper	Fiber	Plastics	Metals	other
Jingkai District	48.3	28.0	10.3	3.4	7.6	2.1	0.3
Lianhu Road	22.3	37.9	6.8	16.5	16.5	0.0	0.0
Yuxiangmen	48.4	22.8	11.4	7.0	8.8	1.6	0.0
Xingqing Road	10.1	56.5	11.3	2.3	14.7	3.4	1.7
Jiandong Road	26.2	46.3	9.5	7.4	9.5	1.1	0.0
Dabaiyang	40.1	29.0	11.2	10.3	7.5	1.9	0.0
Ziqiang Road	28.2	40.6	8.3	9.4	12.5	1.0	0.0
Mean	31.9	37.4	9.8	8.0	11.0	1.6	0.3

**Table 2:** Moisture content and density of MSW in Xi’an city.

Garbage collection site	Jingkai	Lianhu Road	Yuxiangmen	Xingqing Road	Jiandong Road	Dabaiyang	Ziqiang Road	average value
Moisture content (%)	54.4	111.4	75.2	45.6	120.8	84.4	91.6	83.3
Density (g/cm^3^)	0.58	0.41	0.46	0.35	0.38	0.43	0.38	0.43

**Fig 1:**
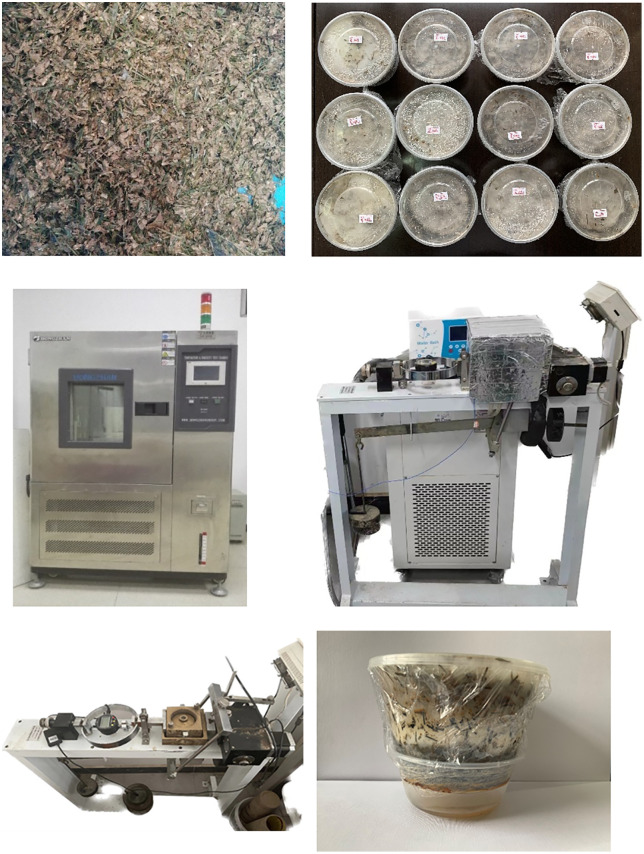
a. Mixed MSW; b. Degraded Samples; c. Environmental testing machine d. Direct shearing machine heat preservation box diagram e. ZJ type strain control type single straight shear instruments f. Gravity Drainage of Leachate.

The samples were made to correctly reflect the true composition of MSW while guaranteeing compatibility with testing devices, based on the preliminary characterization of field-collected MSW samples: MSW samples were kept in an environmental chamber with controlled temperature and humidity to mimic how MSW degradation over time. Biodegradation happened passively in sealed, semi-anaerobic conditions that were similar to a typical landfill. With no extra microbial inoculation; the naturally occurring microbes in the waste were relied to degrade MSW samples over a time periods 190 days. The temperature range of 10°C to 65°C was chosen because studies have shown that microbial and biochemical activity is active in landfills within this range. Following the Standard for Soil Test Method [52] each component soil (32.2%), kitchen trash (37.4%), paper (9.8%), fabric (8.0%), plastics (11.0%), and metal (1.6%) was combined at its natural moisture content. To guarantee moisture equilibration prior to testing all ingredients were completely mixed and were sealed for a full day. Specimens were sieved to <5 mm to ensure uniformity and compatibility with the shear apparatus which limit the mobilization of long fibrous reinforcements. While this can underestimate tensile contributions from long fibers in real landfill scenarios, consistent sample preparation across all conditions enabled reliable comparative analysis of environmental effects on shear strength. This process made sure that the samples accurately represented the hydraulic and structural properties of field MSW [50]. The mixed test sample and degraded MSW samples used in this test are shown in Fig 1a and Fig 1b. Municipal solid waste samples were stably stored in the environmental testing machine (Fig 1c) Shear strength test samples were removed from the environmental testing machine before testing; the direct shear instrument has been modified to accurately control the temperature via a constant-temperature water bath (Fig 1d). To make sure that the temperature of the samples was controlled and monitored correctly, Thermocouples were put in the middle of each MSW specimen to read the internal temperature of the MSW samples. The constant-temperature water bath kept the shear box chamber wall temperature within ±0.5 °C of the target. The sample was preconditioned for at least four hours to maintain a uniform temperature inside the samples. The ZJ-type strain-controlled direct shear apparatus (Nanjing Ningxi Soil Instrument Factory, China), shown in Fig 1e, was used to measure the shear strength parameters of MSW. The device allows precise control of shear displacement and normal load, with a maximum normal stress capacity of 400 kPa and displacement accuracy of 0.001 mm. A typical ZJ-type apparatus has a specimen size of 30 cm², height of 2 cm, and supports lever ratios of 1:12 or 1:20, with shear speeds of 0.02–2.4 mm/min. The instrument was calibrated following the manufacturer’s specifications and periodically rechecked to ensure accuracy.

This shear strength of the samples was periodically measured at four different temperatures (10 °C, 35 °C, 45 °C, 65 °C), five different organic matter contents (15%, 30%, 45%, 60%, and 75%), and five different moisture contents (30%, 80%, 130%, 180%, 230%). Following ASTM D3080, the shear rate of 0.02 mm/min was chosen based on preliminary trials and literature recommendations [50,53] and to ensure fully drained conditions and obtain stable, representative shear strength results for MSW, the samples were preconditioned in perforated containers, allowing gravity drainage of excess leachate into a secondary collection container beneath (Fig 1f). In this arrangement the samples are assumed to reach the equilibrium moisture conditions which represent the field-drained states before testing. Although, pore water pressures were not directly measured, however the test design, drainage setup, and high permeability of the MSW matrix support the validity of the drained assumption. The shear strength sample has a volume of approximately 120 cubic centimeters with diameter of 6.18 cm and a height of 4.0 cm and the mass of each sample was approximately 144 grams. This configuration allowed compatibility with the ZJ-type strain-controlled direct shear apparatus and ensured consistency across all test batches. The void ratio was maintained at approximately 2.2. This corresponds to a porosity of approximately 68.8%, which is consistent with reported values for loose, organic-rich municipal solid waste. Shearing began when the compressive deformation rate of the materials was less than 0.01 mm/h. The selected vertical loads (100, 200, 300, and 400 kPa) and degradation times (15, 30, 40, 70, 100, 130, 160, and 190 days) represent typical ranges for operating landfills. The applied loads correspond to effective cover pressure at depths of approximately 5–20 meters [17] and degradation periods were chosen to capture early, mid, and advanced stages of MSW decomposition based on laboratory and field studies [9]. Based on the degradation age of MSW samples the *c* and *φ* values of each sample were measured in batches. Table 3 lists the test scheme for all 100 samples of this study. Three replicates were used under each test condition and the average results were analyzed. The variation of shear strength parameters (*c* and *φ*) was controlled within ±5% for consistency and reliability of the measurements. Howerver, some limitations may occur in research findings due to complex nature of MSW and uncontrollable factors such as humidity, microbial activity, and sample aging. Also the use of small-scale direct shear tests can limit the full mobilization of tensile reinforcement from long fibrous components in actual field conditions. These factors are acknowledged as constraints in replicating in-situ landfill conditions and should be addressed in future large-scale or in-situ studies.

**Table 3:** Different conditions of the samples prepared in the study.

Sr. No	Moisture content (%)	Organic matter content (%)																			
		15				30				45				60				75			
		Temperature (℃)																			
		10	35	45	65	10	35	45	65	10	35	45	65	10	35	45	65	10	35	45	65
1	30%	S-1	S-2	S-3	S-4	S-21	S-22	S-23	S-24	S-41	S-42	S-43	S-44	S-61	S-62	S-63	S-64	S-81	S-82	S-83	S-84
2	80%	S-5	S-6	S-7	S-8	S-25	S-26	S-27	S-28	S-45	S-46	S-47	S-48	S-65	S-66	S-67	S-68	S-85	S-86	S-87	S-88
3	130%	S-9	S-10	S-11	S-12	S-29	S-30	S-31	S-32	S-49	S-50	S-51	S-52	S-69	S-70	S-71	S-72	S-89	S-90	S-91	S-92
4	180%	S-13	S-14	S-15	S-16	S-33	S-34	S-35	S-36	S-53	S-54	S-55	S-56	S-73	S-74	S-75	S-76	S-93	S-94	S-95	S-96
5	230%	S-17	S-18	S-19	S-20	S-37	S-38	S-39	S-40	S-57	S-58	S-59	S-60	S-77	S-78	S-79	S-80	S-97	S-98	S-99	S-100

To assess the effect of MSW age, moisture content, organic matter content and temperature on the shear strength parameters a series of comprehensive direct shear tests were conducted in controlled laboratory environment. Each parameter was varied individually while keeping other conditions constant o isolate the unique effect of each individual parameter. A combination of statistical and graphical analysis was performed to analyze the experimental data. Graphical analysis of the shear strength data elaborates the variations in *c* and *φ* under different environmental factors of the study. Correlation analysis was performed to assess the direction and degree of correlations between the different factors of study. Automatic linear modeling (ALM) was adopted to forecast *c* and *φ* values under coupled dynamic circumstances and multiple linear regression was employed to evaluate the dependency of shear strength on the study variables. Model performance was evaluated using multiple quality metrics, including R², adjusted R², and root mean square error (RMSE). Residual plots and normal probability plots were examined to confirm the assumptions of linearity and homoscedasticity. Multicollinearity among predictor variables was assessed using variance inflation factors (VIF).

## 3. Results and discussion

### 3.1 Direct shear test results and analysis at different degradation times

**Fig 2:**
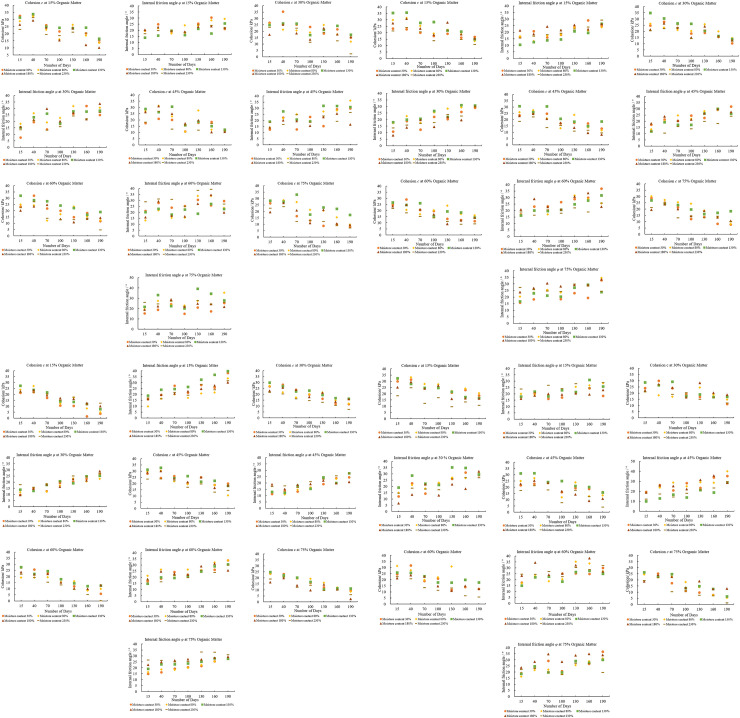
The relationship between the Direct shear test results at different degradation times. **a.** Variations *c* and *φ* over time at different moisture contents, organic matter levels, and at 10°C Temperatures. **b.** Variations *c* and *φ* over time at different moisture contents, organic matter levels at 35°C Temperatures. **c.** Variations *c* and *φ* over time at different moisture contents, organic matter levels, and at 45°C Temperatures. **d.** Variations *c* and *φ* over time at different moisture contents, organic matter levels, and at 65°C Temperatures.

Fig 2a–2d show the resultant relationship curves between the samples’ degradation times and shear strength indices. To assess consistency and variability, three parallel samples were tested under each condition, and the resulting *c* and *φ* values were averaged. The standard deviation for these measurements was consistently below 5%, indicating high repeatability. The results (Fig 2a) have shown a non-linear response to the complicated interaction between moisture and organic matter concentration in determining MSW cohesiveness. The *c* values fall from 32.65 kPa at 30% moisture level to 13.60 kPa at 190% moisture content. On the other hand, at 45% organic matter, the *c* value first increases with moisture, reaching a peak of 31.35 kPa at 80% moisture before falling once again to 11.58 kPa at 190%. The *c* value varies significantly for the maximum organic matter content (75%), increasing to 33.09 kPa at 130% moisture and then falling to 7.21 kPa at 230% moisture. These patterns demonstrate that *c* values are strongly correlated with organic matter and moisture content values.

The moisture and organic matter content of municipal solid waste affects *φ*. When the organic matter content is 15%, *φ* gradually increases over time, reaching a peak of 30.55° after 160 days with a moisture content of 30%. For municipal solid waste with an organic matter content of 30% *φ* typically continues to increase and reach a maximum value of 33.71° after 190 days at a moisture content of 180%. When the organic matter content is 45% *φ* increases significantly over time and reach a peak value of 36.25° after 190 days with a moisture content of 80% indicating that a moderate moisture content can significantly improve the strength of municipal solid waste. The trend continues to 60% organic matter, with a maximum of 39.32° at 160 days and 230% moisture, suggesting that even under high humidity, high organic matter may greatly enhance MSW stability. Similarly, with 75% organic matter, the *φ* reaches 39.27° at 130 days and 130% moisture, demonstrating that MSW with a high organic content retains or even increases its internal friction as moisture and time rise. The *c* values of MSW at 35°C decline over time across all levels of organic matter and moisture content, as shown in Fig 2b. MSW with varied quantities of organic matter and moderate to high moisture content (e.g., 130%) begins with reasonably high cohesiveness, such as 35.42 kPa at 15 days for 15% organic matter and 34.88 kPa for 30% organic matter. But cohesion steadily declines with time, with notable declines shown by 190 days, when *c* has dropped to 15.02 kPa and 13.81 kPa, respectively. This trend suggests that even with high organic content, prolonged exposure to humid environments can reduce the binding strength of municipal solid waste, potentially impacting MSW shear strength over time [54,55].

The *φ* values for MSW at 35°C demonstrate a dynamic interaction between time, moisture content and organic matter content. Depending on moisture content *φ* is lower (10.43° to 21.36°) after 15 days at lower organic matter levels (15%). For a moisture level of 230% *φ* typically rises over time, reaching 29.25° at 190 days. The initial *φ* tends to grow with increasing organic matter content at 45% organic matter, when *φ* values of 18.26° to 24.48° are recorded at 15 and 100 days, respectively. However, high *φ* values were observed at high organic content (60% and 75%) where *φ* approaches 36.85° after 190 days.

Fig 2c illustrates the significant fluctuations in *c* of MSW at 45°C with variations in organic matter, moisture content, and time. MSW with 15% organic matter content exhibits the highest initial *c* at a moisture content of 130% (27.39 kPa at 15 days), but declines rapidly over time, reaching 7.53 kPa at 190 days. MSW with 45% organic matter content exhibits high initial cohesion at all moisture content levels, peaking at 32.76 kPa after 40 days at a moisture content of 130%. However, the *c* gradually decreases at higher moisture contents. When the organic content rises to 60% and 75%, *c* typically decreases more rapidly dropping to almost zero under high moisture content and prolonged exposure. This indicates that while a higher organic content initially enhances the cohesion of the material, long-term exposure to humid and high-temperature environments will significantly weaken its structure over time [56,57]. The *φ* of MSW containing 30% organic matter also increased continuously reaching 28.90° after 190 days at a moisture content of 180%. Over time *φ* values of MSW containing 45% and 60% organic matter also increased significantly. At 130 days age of MSW containing 75% organic matter maintained a high and stable *φ* value, reaching a maximum of 33.28° at a moisture content of 130%. This indicates that high organic matter content is crucial for maintaining the structure and shear strength of MSW under conditions of high temperature and fluctuating humidity.

At the temperature of 65°C the *c* values under different organic matter contents and moisture contents exhibit a clear trend influenced by changes in temperature and humidity (Fig 2d). The cohesive strength of MSW with an organic matter content of 15% was initially high but then varied significantly. At a moisture content of 30% the *c* strength was 29.97 kPa at 15 days and at a moisture content of 80% the *c* strength reached a peak of 31.71 kPa. After 40 days of degradation at a moisture content of 230% the *c* strength decreased to 18.50 kPa. The *c* strength of MSW with an organic matter content of 30% also showed a trend of first increasing and then decreasing. At a moisture content of 30% it reached a maximum of 29.98 kPa after 40 days and then decreased with increasing moisture content. Furthermore, at a moisture content of 30% the *c* strength decreased to 11.20 kPa after 190 days indicating a significant decrease in strength with prolonged exposure time. Compared to municipal solid waste with lower organic matter content, municipal solid waste with 45% organic matter content exhibited higher and more stable *c* values. According to research trends, higher organic matter content provides higher initial strength but is also more susceptible to changes in moisture content. At an organic matter content of 60%, the initial strength *c* was higher, reaching a peak of 31.71 kPa after 40 days with a moisture content of 30%. However, the *c* decreased sharply with increasing moisture content and time; for example, at a moisture content of 230%, the *c* dropped to 5.80 kPa after 190 days. Finally, at a moisture content of 30%, 75% organic matter content showed the highest initial *c* of 25.99 kPa after 15 days. However, *c* dramatically decreases when moisture content rises, particularly above 180%, with a considerable dip to 1.38 kPa at 190 days with 230% moisture. Higher amounts of organic matter often increase cohesiveness, according to the results at 65°C. Time, moisture content, and organic matter content interact to affect *φ* at 65°C. larger organic matter concentration often results in larger friction angles, especially at lower to moderate moisture levels (30%–130%), indicating improved MSW shear strength and stability. While very high moisture (180%–230%) might initially lower *φ* before stabilizing or slightly rising with time, moderate moisture levels usually optimize it. Long-term exposure at this temperature improves MSW stability, as seen by the friction angle’s tendency to rise across all organic matter levels. The following Fig 2a-2d present a complete description of the results and the changing trends of *c* and *φ* for all dynamic conditions of this study for MSW. The interaction between mechanical reorganization and biochemical degradation processes within the MSW matrix is reflected in the temporal evolution of *φ*. Fibrous and plastic components provide tensile resistance and interparticle interlocking in the early phases of degradation, preserving modest *φ* values. Particle fragmentation and settling enhance contact density and decrease voids as decomposition proceeds, gradually increasing *φ*. With extended degradation, the breakdown of fibrous materials and cohesive organic bonds reduces structural interlocking, leading to *φ* stabilizing or slightly declining. These changes indicate that the evolution of *φ* is governed by the balance between compaction-induced densification and decomposition-induced weakening over time. The observed decline in *c* over time is chiefly ascribed to microbial degradation of fibrous organic substances, including paper, food waste, and textiles, which initially facilitate particle bonding and reinforcement. As degradation goes through the hydrolysis and acidogenesis phases, these materials break down into soluble compounds which make the MSW less stable and less cohesive. Simultaneously, partial capillary suction at moderate moisture levels improves bonding and frictional resistance in the early stages of degradation. However, this frictional resistance decreases with the increases of saturation. The temperature between 35 and 45 °C enhance the degradation of organic matter by making microbes work faster. With the start of methanogenesis process the structure settles more and increases friction, this is why *φ* values rise slowly during long-term degradation.

**Fig 3:**
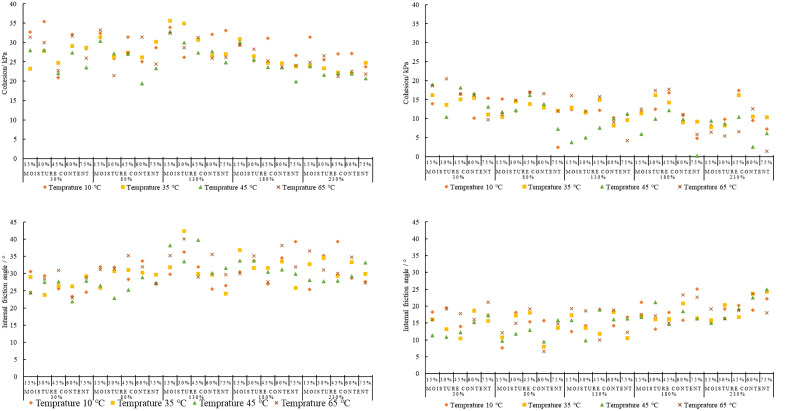
Comparison between the highest and lowest *c* and *φ* values. a. Highest *c* Values Over Time at Different Moisture Contents, Organic Matter Levels, and Temperatures. b. Lowest *c* Values Over Time at Different Moisture Contents, Organic Matter Levels, and Temperatures. c. Highest *φ* values Over Time at Different Moisture Contents, Organic Matter Levels, and Temperatures. d. Lowest *φ* values Over Time at Different Moisture Contents, Organic Matter Levels, and Temperatures.

The highest value of *c* is observed at 35.41 kPa, with 30% organic matter, 30% moisture, and 10ºC. In comparison, the minimum high value is 26.20 kPa at 75% organic matter content, 130% content, and 65ºC. Also, for varying organic matter content and temperature, the highest cohesion values are observed at 130% moisture content. The percentage difference between the maximum and minimum high value is 29.9%. A comparison of the highest *c* values is given in Fig 3a. The lowest *c* values for dynamic conditions are observed at 230% moisture content, but in some situations, it is a minimum at 30%. The minimum *c* 0.17 kPa is observed at 60% organic matter content, 230% moisture content, and 45ºC temperature. Fig 3b shows the relationships among the minimum *c* values across the study’s dynamic conditions. The highest values of *φ* under varying conditions are shown in Fig 3c. Usually, the highest *φ* values are observed at 80% and 130% moisture content. However, in some cases, it is found to have a maximum moisture content of 180% and 230%. *φ* has a maximum value 42.39° at 35ºC temperature, 45% organic matter, and 80% moisture content. The maximum high value of 27.84° is observed at 45ºC, 15% organic matter, and 230% moisture content. The difference between the minimum and maximum *φ* values is 41.4%. At organic matter content of 15% and 10ºC and 35ºC temperatures, *φ* gives a minimum value of 130% moisture content. Fig 3d illustrates how the lowest *φ* values change under various dynamic situations.

The dynamic interactions among moisture, organic matter, and temperature govern MSW shear strength. For instance, MSW with high cohesion (c > 30 kPa) and friction angle (φ > 35°) that was made in conditions of moderate moisture (130%), low-to-moderate organic content (15–45%), and temperatures between 10 and 35 °C may be good for use as backfill or intermediate cover layers. On the other hand, MSW that is very degraded (c < 5 kPa) should not be used for load-bearing purposes or on steep slopes because it is more likely to fail.

### 3.2 Effect of temperature on the shear strength of MSW

**Fig 4:**
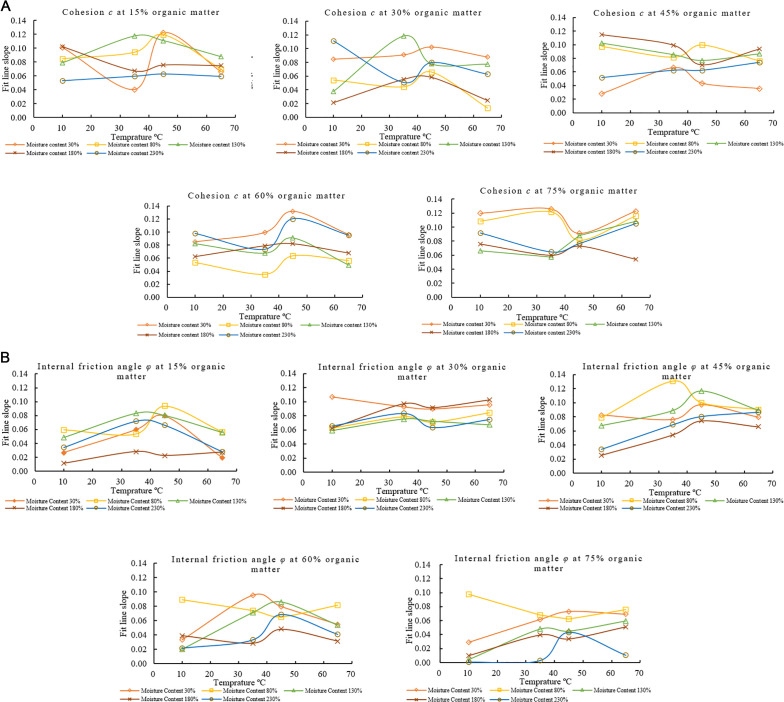
Effect of temperature on Cohesion *c* and Internal friction angle *φ.* **a.** Effect of Temperature on the Fitted Line Slopes of *c* Over Time for Varying Moisture and Organic Matter Contents. **b.** Effect of Temperature on the Fitted Line Slopes of *φ* Over Time for Varying Moisture and Organic Matter Contents.

The slopes of the fitted lines of *c* and *φ* of each sample with time at different degradation temperatures in Fig 4a and 4b are plotted to analyze the relationship law between the sample’s *c* and *φ* at different degradation temperatures. The fit line slope (slope of the fitted regression line) in Fig 4a shows that at lower organic matter content (15% and 30%), *c* generally increases with temperature and moisture content up to a certain threshold. However, it tends to stabilize or decrease at higher moisture content (180% and 230%). As the organic matter content increases (45%, 60%, 75%), *c* shows more complex behavior. At 45% and 60% organic matter, *c* generally peaks at moderate moisture levels (80% to 130%), with some reduction at moisture (230%). Interestingly, at 75% organic matter, *c* values are high across all temperatures at lower moisture contents (30%) and decrease with increasing moisture, significantly beyond 130%. This implies that whereas a high organic matter content may initially promote cohesiveness, it loses effectiveness as moisture saturation rises.

The *φ* increases with increasing temperature when the organic matter content is 15% and temperature is 45°C but decreases significantly at 65°C at low moisture content (Fig 4b). This indicates that the strength of municipal solid waste decreases with increasing material temperature. The enhanced microbial activity and partial decomposition of organic matter at temperatures up to 45°C cause an increase in *c*. Simultaneously, mild heating improves interparticle interactions and reduces excess moisture and significantly increase the *φ*-value. At high temperatures surface roughness and interparticle interactions are reduced which lead the decrease in the *φ*. When the organic matter content reaches 30%, the *φ* tends to be high and less affected by temperature, indicating better stability. However, when the organic matter content reaches 45%, the *φ* begins to decrease with increasing temperature, especially at moisture contents of 180% and 230%; while at lower moisture contents, the *φ* initially increases with increasing temperature. Higher temperatures and moisture contents cause a sharp drop in the *φ* at organic matter contents of 60% and 75%, especially at 65°C and a moisture content of 230%, where the *φ* almost drops to zero. This indicates that when the organic matter content exceeds a certain threshold, especially under high humidity and high temperature conditions, it will reduce the intensity of urban solid waste, resulting in a negative slope for the fitted curve of the *φ* value.

The variation trends of *c* and *φ* across different samples varied with the changing moisture and organic matter contents at different temperatures. This can be due to the complex interactions between thermal effects, moisture migration, and organic matter decomposition within the municipal solid waste. As temperature increases, microbial decomposition of organic matter accelerates and the fibrous and cementing components in the waste decompose which decrease the binding strength. While moderate temperatures between 10°C-35°C may result in partial softening and particle rearrangement temporarily increasing interparticle friction. The samples with comparatively high moisture content where excessive pore water reduces effective stress and leads to a notable decrease in shear strength. Conversely, particle contact is tighter in the samples with lower moisture content and the internal friction angle remains high. This combined effect of temperature, moisture, and organic matter results in different variation patterns among the different test groups. Temperature decreases the shear strength of MSW through physicochemical changes in addition to its effects on microbes’ activity. Heat softens plastic parts and weakens hydrogen bonds in organic matter. It also causes moisture to evaporate which lowers capillary suction and may raise pore pressure. These temperature effects together make interparticle bonding and cohesion weaker, especially when moisture content or temperature is high.

At lower temperatures (10°C–35°C), the moisture film surrounding particles maintains adhesion, and moderate microbial activity enhances bonding through organic by-products, increasing cohesion. At temperature 45°C–65°C thermal softening and accelerates organic degradation and weaken the bonds which reduce c. Temperature influences *φ* by altering particle arrangement and moisture distribution. Moderate temperature enhance compaction and particle contact and slightly increasing *φ*, whereas high temperature causes drying and structural weakening which reduces *φ*. Overall, the thermal sensitivity of organic matter and the dynamic redistribution of moisture govern the temperature-dependent behavior of MSW shear strength. Thermal effects have important engineering implications. Seasonal heating and elevated temperatures in landfills can significantly reduce MSW shear strength which can increase the slope failure risk. Therefore, stability assessments should consider temperature-adjusted shear parameters to maintain safety under varying thermal conditions.

### 3.3 Effect of organic matter content on the shear strength of MSW

**Fig 5:**
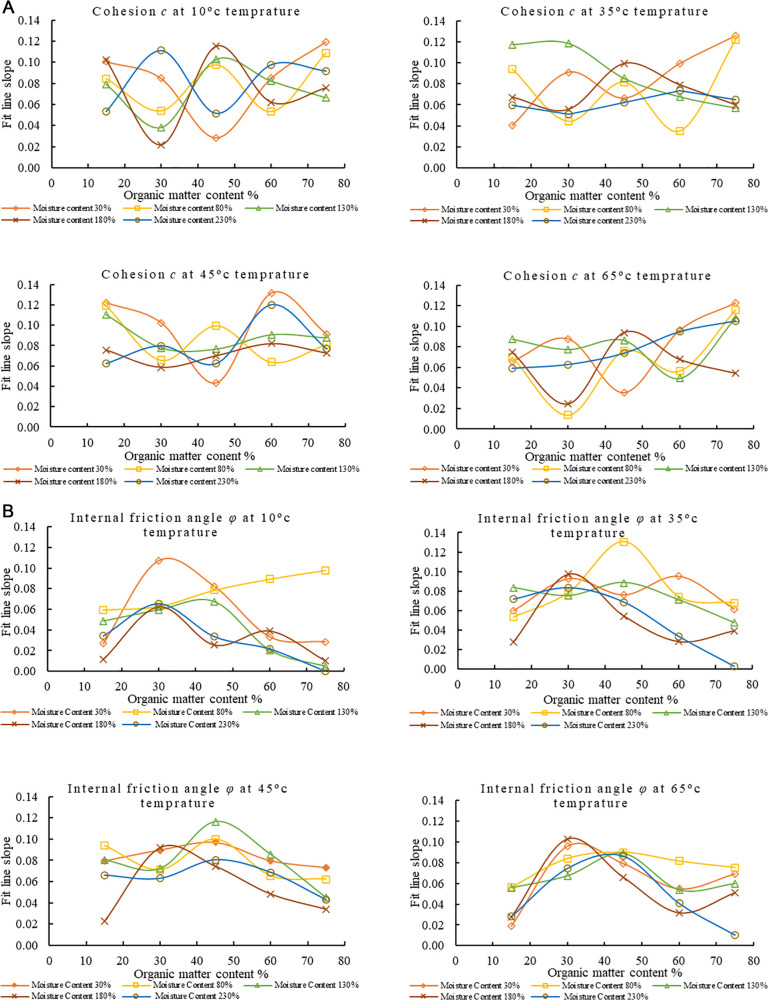
Effect of organic matter content on Cohesion *c* and Internal friction angle *φ.* **a.** Effect of Organic Matter on the Fitted Line Slopes of *c* Over Time for Varying Moisture and Temperatures. **b.** Effect of Organic Matter on the Fitted Line Slopes of *φ* Over Time for Varying Moisture and Temperatures.

The slopes of the fitted lines for *c* and *φ* in Fig 5a-5b were used to analyze the relationship between *c* and *φ* across samples with different organic matter contents. At 10°C, for moisture content at 30%, the fitted line slope shows the highest *c* at 75% organic matter, compared to lower organic matter contents. A similar trend is observed across different moisture levels: higher organic matter content is associated with higher *c* values. For 30% organic matter, *c* peaks at 130% moisture and drops at lower and higher moisture content. The impact on cohesiveness becomes less predictable between 45°C and 65°C. High moisture content might reduce cohesiveness at 65°C. Cohesion values are more stable and exhibit a more accurate response to changes in organic matter and moisture content at low temperatures (10°C); the impact of moisture and organic matter on *c* is more noticeable and stable. Cohesion readings start to fluctuate significantly at a moderate temperature of 35°C. For instance, *c* drops at 30% organic matter and 80% moisture compared to 10°C at the same moisture level, indicating that higher temperatures start to impede the moisture’s capacity to promote cohesion. The temperature effect becomes more noticeable at temperatures between 45°C and 65°C.

Angle of internal of friction *φ* has a non-linear pattern over intervals as organic matter rises. For instance, growing organic matter content at lower intervals, such as 30% moisture content, peaks at 30% organic matter content, and subsequently declines at higher organic matter percentages, such 75% organic matter content. This pattern repeats at other moisture contents, such as 130% and 180%, where *φ* peaks at moderate organic matter levels of 30% or 45% but decreases at higher organic matter levels, such as 75%. At higher moisture contents, such as 230%, *φ* generally declines with increasing organic matter. According to the results, MSW with a high organic content can, over time, maintain or even raise internal friction, especially in environments with moderate moisture content. This pattern suggests that the internal friction angle typically increases, improving resistance to shear and enhancing MSW stability, even if cohesiveness may decrease. Fibrous organic materials, like paper and food waste, first make shear strength stronger by making particles stick together and interlock. Their ability to hold onto water also makes the matrix stick together better and changes the friction between the two surfaces. But as degradation continues, especially at higher temperatures, these organics break down, which makes bonding and structural integrity weaker. This change from making things stronger to making them weaker helps explain why the cohesion and friction angle don’t change in a straight line across different levels of organic matter.

### 3.4 Effect of moisture content on the shear strength of MSW

**Fig 6:**
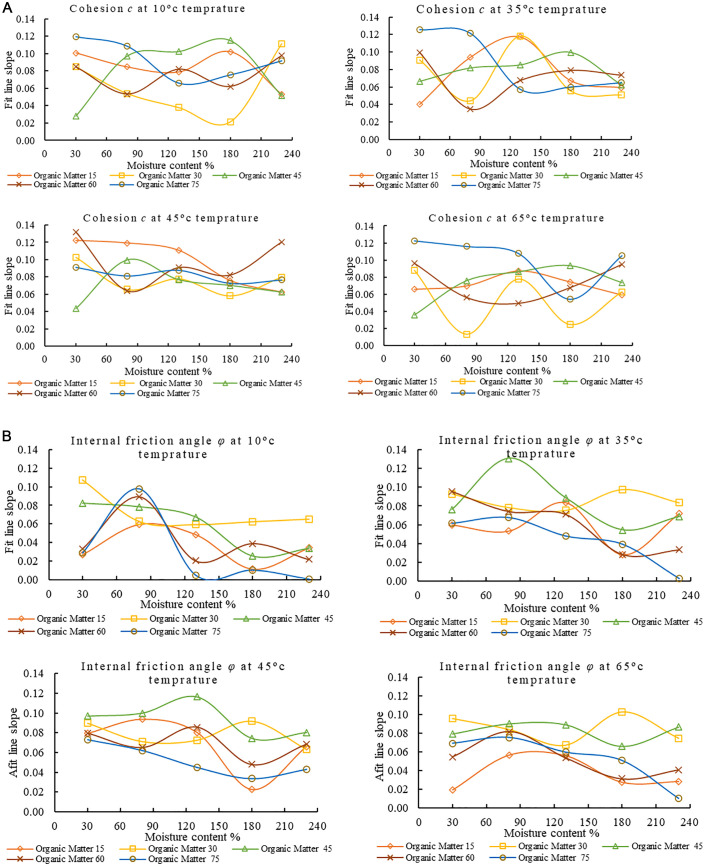
Effect of moisture content on Cohesion *c* and Internal friction angle *φ.* **a.** Effect of Moisture Content on the Fitted Line Slopes of *c* Over Time for Varying Organic Matter Content and Temperatures. **b.** Effect of Moisture Content on the Fitted Line Slopes of *φ* Over Time for Varying Organic Matter Content and Temperatures.

The slopes of the fitted lines of *c* and *φ* of each sample with time at different water contents in Fig 6a-6b were calculated and plotted. In Fig 6a, the percentages of organic matter (15–75%) continue to show varying effects on *c* as moisture content increases (30–230%). At 30% moisture content, *c* generally has higher values. Cohesion has low values at 230% content with different organic matter percentages. However, this pattern is inconsistent across other intervals. For example, at 80% moisture content *c* initially decreases when organic matter content is 15% and 30% and then rises again to 75% organic matter content. At 130% and 180% moisture contents, *c* fluctuates with changing amounts of organic matter contents. Samples with higher organic matter contents of 45% and 60% tend to maintain relatively stable cohesion at different moisture concentrations at 65°C, while materials with low organic matter content exhibit higher fluctuations. At low moisture content (below 50%), the *c* of materials is generally strong, but as the moisture content increases, the *c* of certain components decreases, especially in materials with an organic matter content of 30%. Cohesion is significantly impacted with moisture content percentages, specially at higher moisture and medium organic matter contents (30%–45%). These datasets demonstrate a complex relationship between moisture content and organic matter in MSW, where moisture content typically rises with organic matter but varies over time. It suggests that organic matter has a nonlinear effect on moisture retention in MSW. Increased organic matter content frequently causes *φ* to rise at lower moisture concentrations but at higher moisture contents the impact is less noticeable. For example, MSW with 60% organic matter and 30% moisture content has a greater *φ* than MSW with 15% organic matter. However, the pattern is inverted at 180% moisture content, with MSW with 15% organic matter exhibiting a little higher internal friction angle than MSW with 60% organic matter.

Cohesion has a non-linear relationship with moisture and organic matter concentrations (Fig 6a). Cohesion is improved by capillary bonding and adhesive forces between organic particles at 30% moisture level. However, pore-water buildup and decreased effective stress diminish interparticle bonding when moisture levels rise (beyond 150–230%), which lowers *c*. The local increase in *c* seen at intermediate stages can be explained by partly degraded organics acting momentarily as viscous binders at certain amounts of organic matter.

In contrast, Fig 6b illustrates how lubrication and reduced interlocking cause *φ* to usually decrease with increasing moisture content. However, fibrous and sticky components enhance surface roughness and particle interlocking at intermediate organic matter levels (30–45%), locally raising *φ* even in moist circumstances. However, faster degradation and excess moisture encourage structural softening and pore pressure building at increased organic content (≥ 60%) and temperature, leading to a dramatic drop in *φ*.

Overall, the trends in Fig 6 indicate a complex interaction between moisture, organic matter, and temperature. While excessive moisture and biodegradation reduce the shear strength of municipal solid waste, moderate levels of moisture and biodegradation improve its cohesiveness and frictional resistance.

The changes in *c* and *φ* with moisture and organic matter content at different temperatures reflect the combined hydrothermal-biochemical effects controlling the behavior of urban solid waste. Capillary suction and particle bonding improve *c* and keep stable *φ* at low moisture levels (≤50%) in fibrous organic matter MSW. Moderate moisture (80–130%) gives the best shear strength by making plasticity and bonding better without making the samples too wet. The highest *c* and *φ* values were found in samples with 45–60% organic content. On the other hand, high moisture (≥180%) lowers effective stress and weakens interparticle friction. This is especially true for high organic matter MSW that holds onto extra water and makes the material too weak to be used for structural purposes. These findings identify key moisture thresholds for landfill design. High moisture, especially with high organic content increases slope failure risk while moderate moisture enhances strength making MSW suitable for backfill. Accounting for these effects in design improves landfill stability and safety.

### 3.5 Analysis of shear strength mechanism of MSW

**Fig 7:**
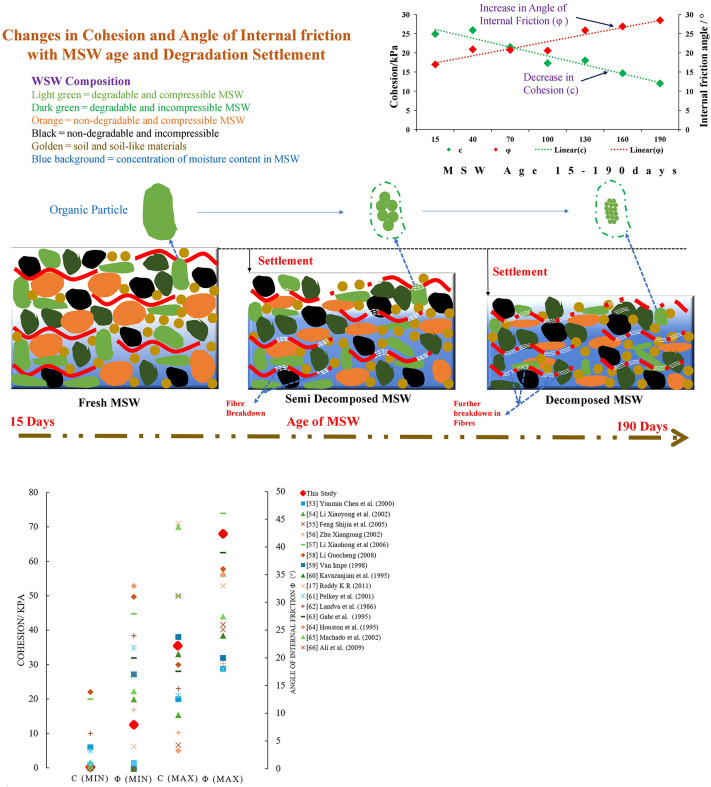
Changes and comparison of *c* & *φ.* **a.** Schematic representation of change in *c* & *φ.* **b.** Comparison of *c* and *φ* values [17,58–71].

The primary cause of the change in the MSW shear strength is the fluctuation of MSW components with age of degradation, the apparent reduction in the proportion of the fibrous components in the garbage is shown in Fig 7a. Fig 7b compares the share values (*c* & *φ*) of this study with those of some previous works, where the high values for *c* & *φ* are higher than those of other researchers, and the low values are comparable to the low values of different research works. The measured ranges (*c* = 0.17–35.41 kPa; *φ* = 7.93–42.39°) are broadly consistent with earlier findings, though minor discrepancies arise from variations in waste composition, sample degradation state, testing methods, and stress conditions. Cohesion values tend to differ most where the organic and fibrous content of MSW is high, while *φ* variations often reflect the proportion of inert and coarse materials. Differences in shear device type (direct vs. triaxial), strain rate, and moisture control further influence the measured parameters. The observed parameters are further influenced by variations in strain rate, moisture management, and shear device type (direct vs. triaxial). The robustness of the observed mechanical behavior is confirmed by the general pattern of diminishing *c* and rising *φ* with gradual degradation.

The shear strength (τ) of MSW consists of three main components: the frictional component $\sigma^{\prime }\text{tan}\varphi_{\text{1}}$, which is proportional to the effective normal stress $\sigma^{\prime }$ acting on the shear surface; cohesive component ($c_{\text{1}}$), generated by direct adhesion, cementation, and electrostatic attraction among the waste particles, and it is independent of normal stress; reinforcing component ($c_{\text{2}}$), which arises from the tensile resistance and interlocking of fibrous materials within the waste mass. These components together define the total shear strength of MSW, as expressed in Equation (i). Mohr–Coulomb assumes a single constant cohesion; the inclusion of $c_{\text{2}}$ accounts for the anisotropic, fibrous structure of MSW, which significantly influences its shear strength and deformation characteristics. The internal friction angle (*φ*) remains associated with interparticle friction and sliding resistance.


$$\tau =c_{\text{1}}+c_{\text{2}}{+\sigma }^{\prime }\text{tan}\varphi_{\text{1}}$$ (i)

The friction $\sigma^{\prime }tan\varphi_{\text{1}}$ the trash soil consists of three main parts: the friction between the fibrous component and the soil particles, the friction within the fibrous component, and the friction within the MSW particles. During the shear process of municipal waste, fibrous components on the shear-damage surface become increasingly stressed as shear deformation progresses. Tensile forces produced by this tension add to the material’s shear strength. The fibrous reinforcement’s tensile strength and the amount of stress it undergoes throughout the shear process are directly correlated with its shear strength. The tautness of the fibrous reinforced phase is closely related to the average principal Stress *σ*_*m*_ in the shear process of MSW. Thus, it is not a fixed constant, expressed as a function of *σ*_*m*_ and shear strain *ε*_*s*_ as shown in the following Equation (ii). Experimental evidence for the relationship between $c_{\text{2}}$, average principal stress (*σₘ*), and shear strain (*εₛ*) was obtained from the observed stress–strain curves of MSW during shear tests. Fibers were found to mobilize tensile resistance as strain increased, enhancing post-peak strength. At moderate *σₘ*, fiber tension and interlocking improved overall shear resistance, whereas at higher strain levels, fiber slippage and breakage led to reduced reinforcement. These trends confirm that $c_{\text{2}}$ varies dynamically with stress and strain, reflecting the reinforcing effect of fibrous components on the overall strength of MSW.


$$c_{\text{2}}=f(\sigma_{m}\;,\;\varepsilon_{s})$$ (ii)

Substituting Eq. (i) into Eq. (ii), the shear strength is expressed as


$$\tau =c_{\text{1}}+f(\sigma_{m}\;,\;\varepsilon_{s})+\sigma^{\prime }\text{tan}\varphi_{\text{1}}$$ (iii)

The Mohr–Coulomb strength criteria theory is frequently used to calculate the shear strength of rubbish, as indicated by the following Equation, as the strength envelope of trash is virtually a straight line:


$$\tau =c+\sigma^{\prime }\text{tan}\varphi$$ (iv)

Comparing Eq. (iii) and Eq. (iv), it can be seen that the cohesive force c in Eq. (iv) is composed of a part of the cohesive force $c_{\text{1}}$ and the reinforcing force $c_{\text{2}}$, the friction force $\sigma^{\prime }\text{tan}\varphi$ in Eq. (iv) contains a part of the reinforcing force and is not entirely the friction strength on the shear surface. Hence, the friction angle *φ* obtained by the calculation of Eq. (iv) is higher than that of the proper friction angle on the shear surface *φ*_1,_ which is on the higher side, and the cohesive force *c* obtained is on the lower side than the actual value.

For the given study, the *c* and φ values can be calculated using the linear equations (v & vi) by performing a linear regression of the changing values of c and *φ* as a function of MSW age and other dynamic conditions of the study. The linear relationships for *c* and *φ* were developed based on their variation with degradation time, as shown in Fig 7a. Although the c and *φ* values were derived from all experimental conditions that incorporated the combined effects of temperature, moisture, and organic matter, the most consistent and dominant trend was observed with respect to degradation time. This is because time determines the process of biochemical decomposition, structural reorganization, and moisture redistribution within urban solid waste, and these processes, in turn, affect its adhesion and friction behavior. Therefore, linear regression was applied to describe the first-order relationship between *c* and *φ* with time, yielding reliable fits within the 15–190-day range. These linear expressions were subsequently incorporated into the improved Mohr–Coulomb model (Equation vii) to capture the time-dependent mechanical evolution of MSW under the controlled conditions of the study.


c = −0.0786x + 27.092 (v)

φ = 0.0624x + 16.619 (vi)

Where x = age of MSW in days

Substituting the *c* and φ from equations (v & vi) in Equation (iv) gives an improved Mohr–Coulomb equation for the dynamic conditions of MSW.


$$\tau =(-\text{0}.\text{0}\text{7}\text{8}\text{6}x\;+\;\text{2}\text{7}.\text{0}\text{9}\text{2})+\sigma^{\prime }\text{tan}(\text{0}.\text{0}\text{6}\text{2}\text{4}x\;+\;\text{1}\text{6}.\text{6}\text{1}\text{9})$$ (vii)

The given time-sensitive model can aid landfill design by predicting strength loss over time under the controlled conditions of the study. It can support slope stability analysis, failure risk assessment, and timing of operations. It should be noted that while the linear relationships between *c*, *φ*, and degradation time provided a strong fit within the 15–190-day range under the controlled condition of the study, the decomposition process of MSW is inherently nonlinear over longer durations. Therefore, these equations are not intended for extrapolation beyond the tested timeframe without further data or advanced modeling, as they may not capture late-stage degradation effects accurately.

## 4 Statistical analyses of the effect of parameters on the degradation of MSW

### 4.1 Correlation analysis

Several researchers [72,73] have used this statistical analysis to forecast the shear strength properties of MSW in the past. The outcomes of the correlation analysis are hereby given in Table 4. The correlation analysis indicates that temperature, moisture content, organic matter, and degradation time exhibit no significant linear relationships, as reflected in their near-zero (0.000) correlation coefficients, suggesting that their interactions with shear strength are non-linear. The results show that cohesion *c* has a negative relationship with these parameters, and increasing values of the dynamic condition’s moisture content, organic matter content, temperature, and age have a decreasing impact on cohesion in MSW. The older age of MSW has a stronger association (.710**), and it has the most significant negative impact on cohesion. The strong negative correlation between cohesion (*c*) and degradation time highlights the loss of interparticle bonding and cementation as organic and fibrous components degrade. As biodegradation proceeds gas and leachate generation alter the internal structure of MSW and reduce adhesive forces and cohesion. The degradation time have a strong negative correlation with *c* (r = –0.71) highlighting it as the dominant driver of cohesion loss. Organic matter and moisture content exhibited moderate negative correlations with *c* (r = –0.29 and –0.18) suggesting their secondary but significant roles. For *φ* both organic matter and degradation time showed moderate positive correlations (r = 0.28 and 0.62), while temperature and moisture had weak correlations. These patterns suggest time-dependent degradation predominantly governs cohesion, while friction angle evolves more gradually under combined influences.

**Table 4:** Correlation analysis.

No.	Parameters	Temperature	Organic matter content	Moisture content	Number of days	*c*	*φ*
1	Temperature	1					
2	Organic Matter Content	.000	1				
3	Moisture Content	.000	.000	1			
4	Number of Days	.000	.000	.000	1		
5	*c*	–.05	–.287**	–.181**	–.710**	1	
6	*φ*	.026	.283**	.018	.622**	–.489**	1

The increase in *φ* can be attributed to the gradual decomposition of organic matter and the redistribution of moisture which improves interlocking and frictional contact between particles. These findings suggest that the dynamic interaction between physical processes (particle rearrangement), chemical processes (bond breaking), and biological processes (microbial activity) controls the evolution of shear strength in municipal solid waste and drives its gradual shift from cohesive to frictional behavior. Similarly, the positive correlation between *φ* and the number of days (r = 0.622, p < 0.01) indicates that as waste decomposes and becomes more granular, frictional resistance gradually replaces cohesion. In summary, these results demonstrate that degradation time plays a decisive role in the mechanical evolution of municipal solid waste, controlling the balance between cohesive loss and frictional increase during aging.

### 4.2 Regression analysis

**Fig 8:**
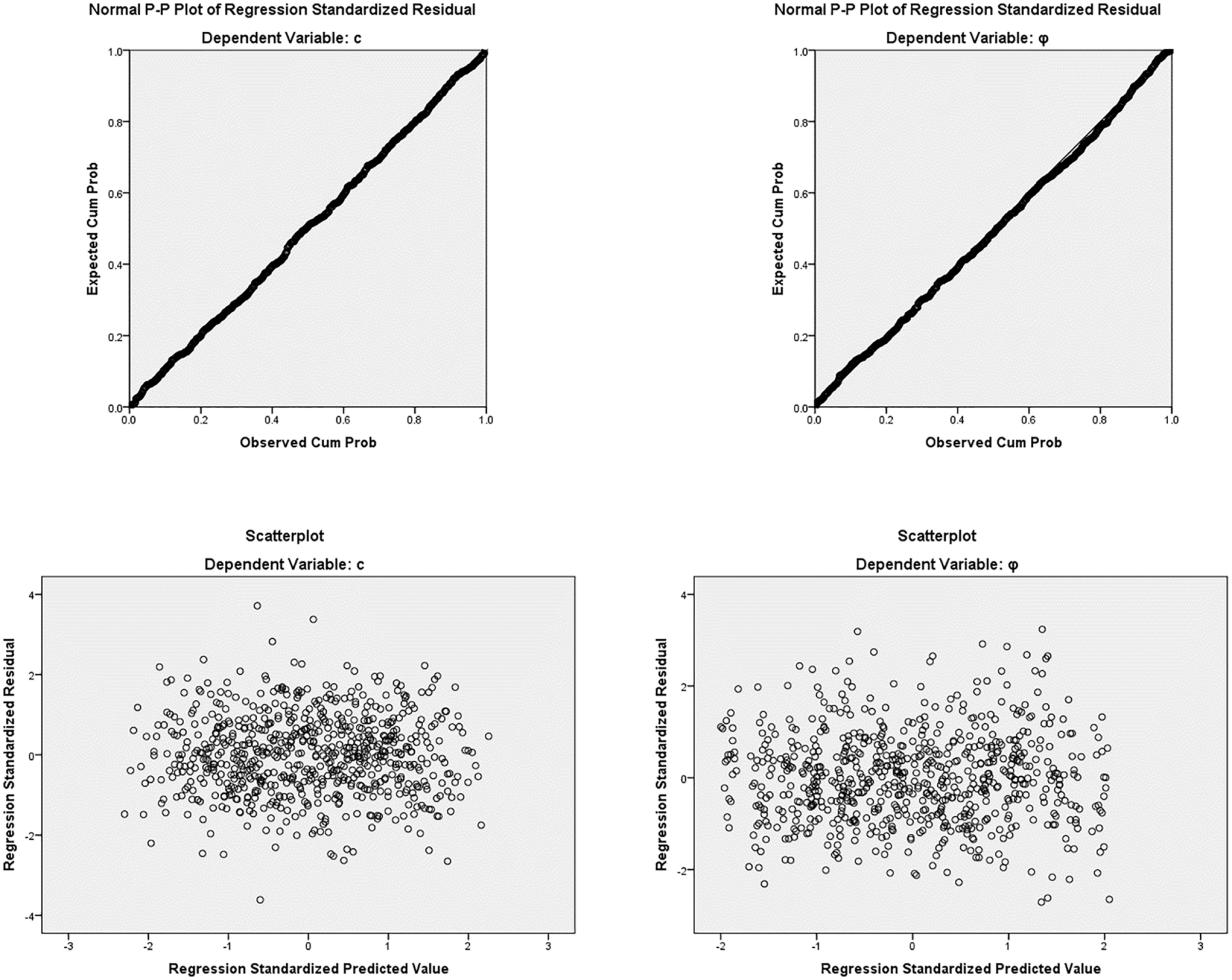
Regression diagnostic plots for c and φ. **a.** Normal P–P Plot **(c)**. **b.** Normal P–P Plot (φ). **c.** Scatterplot Residuals vs Predicted **(c)**. **d.** Scatterplot Residuals vs Predicted (φ).

Several researchers [74–76] have used regression analysis to study leachate characteristics in MSW. This study used regression analysis to assess the statistical influence of the dynamic state of the environmental factors on the shear strength properties of MSW. In regression analysis ordinary least squares (OLS) were used to estimate the coefficients to ensure statistical correctness and repeatability. To evaluate the robustness of the regression framework and confirm the suitability of OLS modelling, model performance indices and diagnostic procedures were conducted for both cohesion c and friction angle *φ* models. The multiple linear regression model for cohesion c demonstrated strong explanatory performance (R = 0.789, R² = 0.622, Adj. R² = 0.620) with a standard error of estimate of 4.027, and the overall model was statistically significant (F = 286.164, p < 0.001). Similarly, the friction angle *φ* model showed good predictive capability (R = 0.684, R² = 0.468, Adj. R² = 0.465) with a standard error of estimate of 4.331, and was also highly significant (F = 152.675, p < 0.001). Multicollinearity was assessed using tolerance and variance inflation factor (VIF) statistics; all predictors exhibited Tolerance = 1.000 and VIF = 1.000, confirming negligible multicollinearity and stability of regression coefficients. Independence of residuals was evaluated using the Durbin–Watson statistic (1.436 for *c* and 1.505 for *φ*, indicating no severe autocorrelation. Model influence diagnostics further confirmed robustness, as Cook’s distance values were minimal (maximum Cook’s distance 0.018 for c and 0.019 for *φ*, suggesting no influential outliers. Residual diagnostic plots further supported the validity of the OLS assumptions: the Normal P–P plots of standardized residuals showed points closely aligned with the diagonal reference line, indicating approximately normal residual distributions (Fig 8a and 8b), while the standardized residuals versus standardized predicted value scatterplots (ZRESID vs ZPRED) displayed randomly dispersed residuals around zero without systematic curvature or funnel-shaped patterns, supporting linearity and approximate homoscedasticity (Fig 8c and 8d). Overall, these diagnostics confirm that OLS regression provides a statistically appropriate and credible modelling approach for relating shear strength parameters to environmental variables in the MSW dataset. Although the adopted linear relationships between degradation time and shear strength parameters yield a practical fit within the 15–190-day range, it is crucial to acknowledge that long-term MSW decomposition is fundamentally non-linear. Biological and structural changes may speed up or slow down outside of this window, and it is important to be careful when using the linear model outside of the tested period.

#### Dependence of *c* on the predictors.

The regression analysis in Table 5 provides insights into the relationships between the dynamic conditions (predictors) and *c* values. A significant correlation exists for pH values, with R = .789 and F = 286.164, suggesting that the overall model effectively predicts cohesion values. However, the negative sign of the Beta (β) Coefficient and negative t–values indicate a negative impact of predictors on cohesion values. The p–values for all predictors are less than 0.5, indicating acceptable relationships.

**Table 5:** Regression analysis for *c.*

No.	Regression weights	Beta (β) coefficient	R	$R^{2}$	F	t–Value	*p*–Value
	Costant	33.984				56.474	.000
1	Temprature→*c*	–.019	.789	.622	286.164	–2.495	.013
2	Organic Matter Content→*c*	–.088				–12.296	.000
3	Moisture Content→*c*	–.017				–7.778	.000
4	Number of Days→*c*	–.079				–30.442	.000

The general regression equation (v) for this model is given below:


$$y=\;\alpha +\beta_{\text{1}}X_{\text{1}}+\beta_{\text{2}}X_{\text{2}}+\beta_{\text{3}}X_{\text{3}}+\beta_{\text{4}}X_{\text{4}}$$ (viii)

The Table 5 shows that all predictors, Temperature, Organic Matter Content, Moisture Content and number of days, have p–values < 0.5, suggesting they are significant predictors in this model.

Given this information, the regression equation (vi) would be, with the value of alpha constant (33.984):


$$c=\;\text{3}\text{3}.\text{9}\text{8}\text{4}+\left(-.\text{0}.\text{19}\times X_{\text{1}}\right)+\left(-.\text{0}.\text{88}\times X_{\text{2}}\right)+\left(-.\text{0}\text{1}\text{7}\times X_{\text{3}}\right)+(-.\text{079}\times X_{\text{4}})$$ (ix)

Where X_1_ = Temperature, X_2_ = Organic Matter Content, X_3_ = Moisture Content and X_4_ = Number of days.

Equations (ix) can be used to predict cohesion values for future works based on the dynamic conditions of this study.

#### Dependence of *φ* on the predictors.

Table 6 shows that MSW’s organic matter content age shows statistically significant relationships with *φ*, as indicated by *p*–values (all < 0.05) and a high F–value of 152.675. Also, the R–value of 0.684 and the R^2^–value indicate a strong relationship and Dependence of *φ* on the predictors. The *φ* is positively influenced by organic matter and the number of days. This behavior is consistent with MSW’s mechanical development during degradation. The *p–v*alues for moisture content and temperature are greater than 0.05, so they are considered insignificant in this relationship. The regression equation would be formed for *φ*, with the value of alpha constant (33.984):

**Table 6:** Regression analysis for *φ.*

No.	Regression weights	Beta (β) coefficient	R	$R^{2}$	F	t–Value	*p*–Value
	Constant	12.567				19.419	.000
1	Temprature→φ	.008	.684	.468	152.675	.929	.353
2	Organic Matter Content→φ	.079				10.235	.000
3	Moisture Content→φ	.002				.664	.507
4	Number of Days→φ	.062				22.464	.000


$$c=\;\text{1}\text{2}.\text{6}\text{5}\text{7}+\left(.\text{079 }\times X_{\text{2}}\right)+(.\text{062}\times X_{\text{4}})$$ (x)

Equations (x) can be used to predict *φ* for future works based on this study.

### 4.3 Automatic Linear Modeling (ALM)

**Fig 9:**
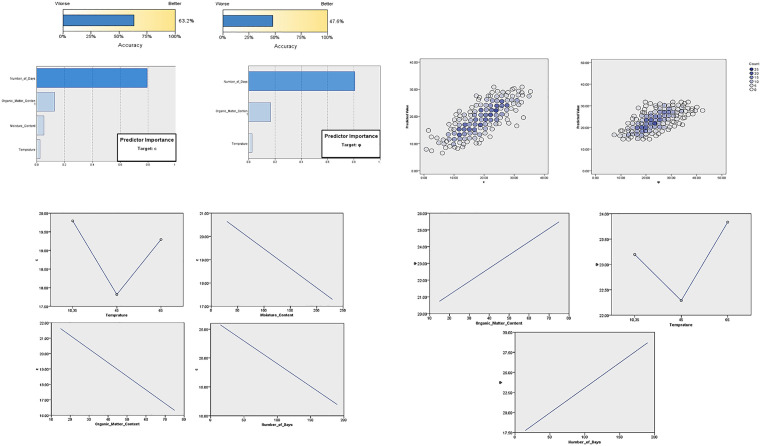
Automatic linear modeling. a. Predicted data for the model a) *c* **b)** φ. **b.** Predictor’s importance in the estimating models i) *c* **ii)** φ. **c.** Discarded scatterplot of observed vs predicted values for i) *c* **ii)** φ. **d.** Estimated Mean Values for *c*: Automatic Linear Modeling. **e.** Estimated Mean Values **(pH)**: Automatic Linear Modeling.

ALM is applied to further study the influence of predictors, as the dataset is complex and contains 700 distinct relationships between the parameters. In the proposed model, the impact of predictors on MSW degradation characteristics was analyzed using the ALM method. The model fit structure for the Dependence of *c* and *φ* shows that $R^{\text{2}}$. The values are 63.2% and 47.6%, respectively, as shown in Fig 9a. The model summary in Fig 9b shows the predictor importance charts; for cohesion *c* values, the number of days has the highest importance (0.79), indicating it has the most significant influence on the predictive model. The other predictors are less critical in predicting the *c* values. The scatter plot in Fig 9c displays data values on the x–axis and predicted values on the y–axis. It shows that more samples are at 45°, and the models were quite precise. Fig 9d consists of the estimated means charts representing and analyzing the comparison between the mean values of the dependent variable (*c*) across different levels or categories of the predictor’s parameters. Fig 9e represents the estimated mean charts for φ values. The predicted ALM model has suggested a gradual increase in φ value with the rise in the age of MSW. Similarly, the φ values will have an increasing trend with the increasing organic matter content. For temperature, it first shows a decreasing trend up to 45ºC, then a rising trend. Exploratory data analysis revealed that moisture content exhibited a non-linear relationship with the shear strength parameters (*c* and *φ*), with an initial increase in strength at moderate moisture levels due to capillary bonding, followed by a sharp decline at higher moisture levels due to saturation and reduced effective stress. This non-monotonic trend violated the linearity assumption required by the Automatic Linear Modeling (ALM) framework; therefore, moisture content was automatically excluded from AIC-based variable selection because it was statistically insignificant (p > 0.05).

Fig 8d and 8e shows that the temperature, organic matter content, and degradation time significantly affect the shear strength of municipal solid waste (MSW). The model achieves an R² value of 63.2% for cohesion (*c*) and 47.6% for *φ*. Evaluation metrics such as root mean square error (RMSE) and corrected R² validate that the ALM model provides a reliable fit without significant overfitting. ALM offers valuable linear approximations; however, it fails to encapsulate intricate nonlinear trends, especially the moisture-shear strength relationship identified in this study. Although moisture content was excluded from the ALM model due to its lack of linear significance but it does not imply physical irrelevance as previous sections demonstrate that has nonlinear influence on shear strength. Its statistical exclusion reflects model structure limitations rather than true engineering insignificance. Future research could be enhanced by employing more adaptable models, including polynomial regression, generalized additive models (GAM), or machine learning techniques (e.g., random forest, support vector regression). These models can show nonlinear interactions better, but they are more complicated.

The combined hydro-biochemical interactions inside MSW are reflected in the non-linear fluctuation of cohesion (*c*) and internal friction angle (*φ*) with moisture, organic matter, and temperature. Thin water films and capillary tension promote bonding and particle interlocking at moderate moisture levels which raise *c* and *φ*. After a certain point, too much moisture decreases frictional resistance and effective stress, which lowers shear strength. Organic matter initially promotes cohesion through adhesion and the reinforcing effect of fibrous components. However, with the increase in temperature the structural elements break down due to biodegradation and reduces *c* and *φ*. The rise in temperature causes the nonlinear mechanical behavior in this process by accelerating microbial activity and alters pore water distribution. At low organic matter contents interparticle bonding is limited and results in moderate frictional resistance. At moderate level (30–45%) fibrous and sticky organic matter enhances interparticle interlocking and cohesion and raise the *φ* value. Excessively high organic matter content (75%) causes deformability and pore water retention and reduce the frictional contact area and effective stress. Increased temperature and humidity intensify lubrication and degradation effects which accelerate the loss of fiber-reinforced materials and reduce the *φ* value. This nonlinear trend indicates how hydrothermal-biochemical interactions in MSW matrices and achieve structural reinforcement at moderate organic matter concentrations and lead to structural softening or weakening at higher concentrations.

The results indicate that the combined effects of degradation age, moisture content, organic matter content and temperature determine the shear strength behavior of MSW. Effective stress and particle adhesion are modulated by moisture and organic matter content, while the age of the MSW significantly alters the structure and bonding patterns of the waste. Temperature indirectly affects mechanical responses and accelerates the degradation process. The stability and deformation characteristics of MSW are jointly determined by these interactions, highlighting the necessity of considering the coupling of environmental and compositional factors in waste geomechanically studies.

## 5 Conclusions

To investigate the shear strength characteristics of municipal solid waste, 100 samples were observed under 700 different dynamic conditions. Data analysis showed that age, temperature, moisture content, and organic matter concentration all affect the geotechnical engineering properties of municipal solid waste. According to the test findings, cohesiveness is greatly reduced by high temperatures (45°C–65°C) and excessive moisture (180%–230%), while it is much enhanced by moderate moisture levels (80%–130%) and lower temperatures (10°C). The high *φ* values can be achieved at (80–130%) moisture content, (45–60%) organic matter, and by keeping the temperature between (10–35°C). For the high *c* values, 130% moisture content, (15–30%) organic matter, and (10–35°C) temperature are recommended. This study shows that the shear strength of municipal solid waste is affected by a variety of interacting factors, among which the age of the MSW has the most significant impact, followed by moisture content, organic matter content, and temperature.

To achieve maximum shear strength (*τ*), the optimal parameters for municipal solid waste (MSW) are recommended as follows: moderate moisture content (130%), organic matter content between 45% and 60%, and temperature range of 10°C to 35°C. Regression and correlation analyses show that MSW age is the most significant negative factor affecting its cohesiveness. The friction angle is positively correlated with MSW age and organic matter content. Overall, the results reveal the complex relationship between environmental factors and the geotechnical properties of MSW, providing crucial data for waste management practices and landfill stability in practical engineering applications.

The findings of study suggests that the controlling moisture is the most important to improve the shear strength of MSW because too much water makes cohesion and friction much weaker. After that, the amount of organic matter should be controlled to stop too much softening during degradation, and the temperature should be controlled in bioreactors or landfills. This study offers valuable insights on how environmental factors have coupling effect on the shear strength parameters of MSW; however, several limitations must be recognized. The direct shear test imposes a predefined shear plane and uses relatively small specimens, which may not fully represent the heterogeneous structure, complex stress paths, and strain localization of in-situ landfill waste. Drainage conditions were not directly monitored (e.g., via pore-water pressure), and therefore fully drained behavior was assumed rather than verified. In addition, due to fibrous reinforcement within MSW, the obtained cohesion c and friction angle *φ* should be interpreted as apparatus-dependent composite strength indices rather than fundamental material properties. Furthermore, the regression-based correlations (ALM/OLS) were calibrated from laboratory data and may not fully capture field-scale variability, coupled hydro-biochemical degradation effects, and potential nonlinear relationships. Future studies should employ larger-scale or advanced shear testing (e.g., ring shear/true triaxial), incorporate direct pore-pressure, validate models using independent laboratory and field datasets, and further explore nonlinear predictive approaches such as polynomial regression and generalized additive models (GAM) to improve generalizability and predictive reliability.

## Supporting information

S1 File Graphical abstract. (TIF)

S2 File Highlights. (DOCX)
