# Supplementary figures and images for: Experimental and coupling analysis of municipal solid waste (MSW) shear strength under multiple influencing parameters
Source: PLoS One. 2026 Mar 5;21(3):e0344191. doi: 10.1371/journal.pone.0344191 (PMC12962542; doi:10.1371/journal.pone.0344191)

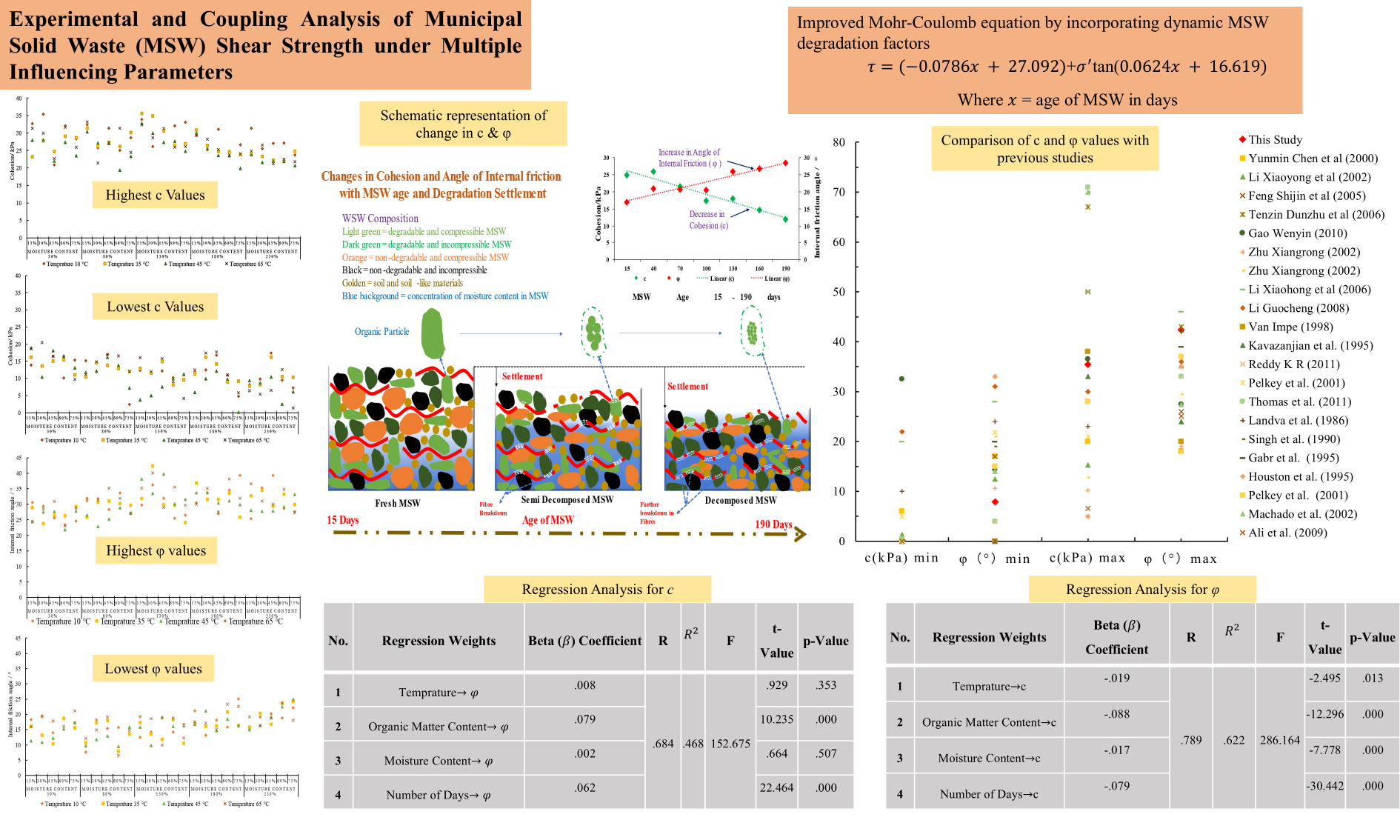

Supplement: S1 File — (TIF) [file pone.0344191.s001.tif]
