# Supplementary material for: Experimental and coupling analysis of municipal solid waste (MSW) shear strength under multiple influencing parameters
Source: PLoS One. 2026 Mar 5;21(3):e0344191. doi: 10.1371/journal.pone.0344191 (PMC12962542; doi:10.1371/journal.pone.0344191)
Supplement: S2 File — (DOCX) [file pone.0344191.s002.docx]

**Highlights**

- The study carries out a multi-factor coupling analysis of factors affecting MSW Shear Strength.
- Highest φ values are achieved with 80-130% moisture, 45-60% organic matter, and between 10-35°C.
- For high c values, 130% moisture, 15-30% organic matter, and 10-35°C temperature are recommended.
- The Mohr-Coulomb equation is improved by incorporating dynamic MSW degradation factors.
- Multiple linear regression equation and ALM model are proposed to predicted c & φ values.
